# A Contribution to the Therapeutics of Iron

**Published:** 1897-11

**Authors:** 

**Affiliations:** Assistant Physician to the Private Gynecological Clinic of Dr. Mackenrodt, Berlin


### Selections and Abstracts.

### A CONTRIBUTION TO THE THERAPEUTICS OF IRON.

BY DR. GELLHORN,

Assistant Physician to the Private Gynecological Clinic of Dr. Mackenrodt, Berlin.

The skeptical assertions of Dr. Bunge, regarding the value of ferruginous medication, at the Congress for Internal Medicine of 1895, evoked an almost unanimous and vigorous opposition in the discussion which followed the reading of his paper. The doubts expressed by him in reference to an insufficient absorption of the inorganic preparations of iron could at that time only be controverted, in the main, by the results of practical experience derived from the administration of iron. However, Quincke even then pointed out that his investigations on the subject, which had not yet been concluded, had demonstrated the absorption of iron preparations given for medicinal purposes, and their utilization in the body. In 1896, at the Congress for Internal Medicine, Quincke reported the results of his experiments which meanwhile had been completed, and which confirmed in every respect the above-mentioned statement. He had made it his aim to trace the course of iron along the intestinal canal, by means of micro-chemical reactions, and for this purpose fed white mice for a number of days with cheese, to which had been added various ferruginous preparations. The animals were killed during feeding, or after the lapse of a certain interval, and the viscera, especially the intestinal canal, hardened in alcohol, cut open and examined for the presence of iron with sulphide of ammonium as a reagent. It was thus found that iron is absorbed exclusively in the duodenum, and this applies both to the iron in the food and that administered medicinally. It was detected in the duodenal epithelium and in the stroma of the villi, and is visible even to the naked eye. Furthermore, iron is found deposited especially in the liver cells, in a form perceptible on microscopical examination, and in rare cases could be detected by microscopical means in the cortical tubules of the kidneys.

These investigations of Quincke have demonstrated incontestably that the favorable results which have been obtained, since olden times, from the administration of iron are actually attributable to its absorption, and not, as Bunge would have it, to accidental circumstances, to diet alone, or even suggestion. Control experiments in this direction with indifferent medicaments are readily carried out, and were repeatedly mentioned at the Congress of 1895. It should be added that these control experiments were followed by no change, or only by a transient improvement in the condition of the patient.

At the last Congress for Internal Medicine, the subject of the therapeutics of iron was so thoroughly ventilated by the foremost clinicians, as well as by numerous physicians in late years, that a new contribution would appear superfluous. This subject, however, is of such immense importance to the general practitioner, that a cumulation of material is necessary in order to eliminate the least doubt as to the efficacy of a therapeutic measure which, originating at first on the basis of speculation, and later supported by the results of empirical observations, has finally been demonstrated to be of value by exact experimentation.

In the following I will only discuss the clinical aspects of this question. I was encouraged in undertaking this work by my honored teacher, Dr. Mackenrodt, who has assisted me in every possible way. In the management of chlorosis and anæmia, and the host of sequelæ of these diseases, the physician would be powerless if he had not in iron a specific, or at least a potent and indispensable adjunct to his other therapeutic resources. The patients, who belong for the most part to the working classes, give in main the same group of symptoms: amenorrhœa, scanty or profuse, weakening, irregular, usually premature, menses; headache, anorexia and dyspepsia; neuralgias, and almost invariably marked lassitude, which interferes markedly with their ability to work. In these cases prompt and radical help must be afforded, in order to restore to the patients their full working capacity as soon as possible. It is well known that the therapeutic value of the various iron preparations differs greatly. This is shown *a priori* by the abundance of manufactured products of this kind. My experience relates chiefly to three preparations, pilulæ chinini cum ferro, formula magistralis of Berlin, liquor ferri albuminati, and the neutral Pepto-Mangan (Gude). My results with the first of these three remedies have been very indifferent, while with the liquor ferri albuminati of the pharmacopia they were somewhat better. I have instituted accurate examinations, however, with only Gude's Pepto-Mangan, and the data given further on relate to this remedy alone. Owing to my limited experience with the many other preparations employed by various authors, I would not designate the Pepto-Mangan as a universal remedy, or as the only efficient preparation.

Still another remark: There can be no doubt that our medical intervention, no matter of what kind, is materially assisted by psychical impressions. This applies especially to our female patients, who are extremely susceptible to mental influences of this character. Hence, it may readily occur at the commencement of treatment that the previous disorders are less strongly felt, and it is therefore unfortunate that an objective criterion for the existing improvement is not at our disposal, as such we would regard regular examinations of the quantity of hæmoglobin in the blood. In the observations reported, these were made with Gower's hæmoglobinometer. This instrument is very convenient, and is superior to Fleischl's apparatus for the use of the general practitioner, especially on account of its cheaper cost. The tests are very exact; any existing errors are the less to be considered since they occur uniformly and in about the same degree during the entire course of the experiments.

That dietetic treatment alone may be successful in anæmic and chlorotic patients was laid down as a dictum by Immermann and Reinert at the Congress for Internal Medicine of 1895. It is natural to suppose that poor and ill-nourished persons would gain in strength under the influence of a proper and invigorating diet; nevertheless after eight to fourteen days a cessation in the improvement occurs and the old disorders return. These authors, as well as Nothnagel and v. Ziemssen, consider an invigorating diet as only a valuable adjunct; both of the latter, moreover, regard rest in bed for several weeks as an important factor in the cure. Since several years Mackenrodt has also instituted a large series of observations of this kind, not yet published, in which, for purposes of control, he employed quantitative estimation of the hæmoglobin. It was found by him that under the influence of hygienic and dietetic regulations alone the quantity of hæmoglobin in the blood increased only at the commencement of treatment, and then only in a dilatory manner.

In the case of one of my patients I proceeded as follows: I prescribed Pepto-Mangan (Gude), one teaspoonful three times daily after meals, and regulated the diet in accordance with the directions furnished with the preparation. Sour and fatty foods, as well as raw fruits, are to be avoided under all circumstances, Fritsch (Diseases of Women, 1892, p. 469) advises, indeed, that the desire for acids manifested by chlorotics should be gratified. According to my experience, however, this craving for acids is to be regarded as a pathological condition of the alimentary tract, which is made worse by further supply of acids, but can be successfully overcome by an unstimulating diet. In cases where the social conditions in any way permitted, I allowed the patient to take a small glass of red wine three times daily, but never during a period of one hour before and after the administration of the medicament, in order to prevent the combination of the tannic acid contained in the wine with the iron. The use of potatoes was restricted as much as possible, at least during the first four weeks. Furthermore, I resorted to the dietetic regulations customary in these cases, but changed them to advantage when, as so often happens, obstinate constipation was present, following in this respect the suggestions of Hebra, which have recently been again advocated by Ruge (Transactions of the Obstetrical and Gynecological Society of Berlin, 13, III., 1896), and obtained generally excellent results. In contrast to several authors who made it a practice to remove any existing dyspepsia before resorting to the use of iron, I have followed the method of v. Ziemssen and Baumler, of at once administering iron—unless the presence of a severe gastric affection, especially ulcer of the stomach, could be positively determined—and observed as early as the end of one or two weeks an increase of appetite and subsidence of the gastric disorder.

I would lay especial stress upon systematic exercise in the open air. I orderd the patients, who, with but two exceptions, were treated out of bed, to take a stroll at midday, at first of five to ten minutes' duration. At the end of three to four days they were allowed to remain outdoors for five to ten minutes longer.

After each walk they were advised to take off their corsets, put on their slippers, and rest for an hour on the sofa. Under this treatment, the lassitude invariably vanished after a time.

In the manner thus described I have treated in all about sixty patients. In twenty-four cases I instituted quantitative estimations of hæmoglobin at regular intervals of three, five or eight days. Under normal conditions the quantity of hæmoglobin in women amounts to 12 59 per cent. when estimated in comparison with the other constituents of the blood. Among my cases the lowest amount met with was in a single instance, 30 per cent. of the normal, that is to say, of the above 12.59 per cent. Next to this was the following case with 32 per cent. of the normal:

Miss G. W., twenty-two years old, seamstress, related that she had been under treatment for four years for chlorosis. Since the age of nineteen her menses had been scanty, occurring before the usual time, and of three to eight days' duration. On September 26, 1895, a *remotio secundinarum* occurred after an abortion induced in the fourth month. At present she complains of darting pains in the upper portions of the lungs, headaches, and rapid loss of strength.

January 9, 1896, anæmic appearance; physical examination, especially of lungs, negative. Quantity of hæmoglobin, 32 per cent. Ordered Pepto-Mangan (Gude), diet, etc.

January 13, considerable improvement of the general condition. Hæmoglobin, 45 per cent.

January 17, since previous day, diarrhœa, due to gross errors in diet, troublesome eructations. Ordered tinct. opii, 15 drops three times daily. Hæmoglobin, 47 per cent.

January 21, improved after use of tinct. opii, no more gastric pains or eructations. Headaches have completely disappeared, lassitude less marked. Hæmoglobin, 55 per cent.

January 31, condition unchanged, ceased menstruating on previous day, the flow having lasted five days.

February 8-28, patient feels well and no longer complains of pains in the lungs. Appetite and bowels regular. Hæmoglobin, constantly 55 per cent.

March 5, no change. Hæmoglobin, 62 per cent.

March 11, hæmoglobin, 68 per cent. March 27, 77½ per cent.

Unfortunately, as in most of these cases, the patient's visits ceased as soon as she felt entirely capable of going to work. As a matter of fact, the increase of hæmoglobin in this case was tardy, as in four other cases in which the quantity at the beginning was 34, 35, 37, and 38 per cent. of the normal. In eighteen other instances in which the initial amount was higher, viz., 42-75 per cent. of the normal, progress was more rapid as a rule.

This is most strikingly illustrated in the following case:

Miss C. B., aged fifteen years, complains of violent headaches, visual disorders, loss of appetite, a feeling of pressure over the stomach, constipation, and general lassitude.

June 2, 1896, *status præsens*: mucous membranes pale; physical examination, negative; heart, normal; quantity of hæmoglobin, 45 per cent. Prescribed as in above case.

June 9, headache has disappeared; condition otherwise unchanged. Hæmoglobin, 45 per cent.

June 16, improvement. Hæmoglobin, 51 per cent.

June 23, decided improvement. Hæmoglobin, 55 per cent.

July 8, patient free from complaints; cheeks ruddy, lips and conjunctiva red. Hæmoglobin, 78 per cent.

July 23, and September 24, continued good health.

I also derived exceedingly favorable results from the use of Pepto-Mangan, (Gude), in patients who came to us for operations after having been exhausted by protracted hemorrhages. Of course convalescence in such cases is delayed; the system recuperates but slowly from the double injury inflicted by the losses of blood and the operative intervention. Digestive disturbances are especially apt to be troublesome. In these cases ferruginous medication often produces remarkable improvement.

I can not close this paper without calling attention to the beneficial influence exerted by Pepto-Mangan, (Gude), in anæmic neuralgias, and as an illustration of its effects in this class of cases add in brief the following history of a case:

Mrs. K., aged thirty-five years, very pale and ill-nourished, suffers from intercostal neuralgia on the left side.

January 30, 1896, quantity of hæmoglobin, 68 per cent. of the normal.

February 5. In the meantime has suffered on two days with violent headaches. Intercostal neuralgia persists. Appetite good; no gastric disturbances. Hæmoglobin, 69 per cent.

February 12, no longer troubled with headaches with exception of one attack of neuralgia in the area supplied by the left supra-orbital nerve. The paroxysms of pain on the left side of the chest have become less frequent. The lassitude has subsided. The mucous membranes are still anæmic. On the whole, the patient feels better and more vigorous than before the commencement of treatment. Hæmoglobin, 75 per cent.

February 18, considerable improvement of neuralgias; no headaches, nor digestive disturbances. General health improved. Menses appear earlier than previously, this being the second day of the flow. Hæmoglobin, 73 per cent.

February 26. During the preceding days transient deterioration of her conditions owing to mental excitement. Menstrual period has been normal. Hæmoglobin, not estimated.

March 2, patient no longer complains. Intercostal neuralgias have ceased to occur except on rare occasions. Hæmoglobin, 76 per cent. March 13, health good in general. Iron discontinued on account of gastric disturbances which are said to result from excitement. Ordered strict diet and iron to be resumed.

March 19, complete restoration of health. Hæmoglobin, 82 per cent.

That the final estimates did not yield the normal quantity is not surprising since it is frequently somewhat reduced even in healthy persons. At any rate, the objective and subjective state of the patients in the above cases, as well as in the others not reported in detail, afforded the impression that a radical cure with complete restoration of the ability to work has been effected.

It must be conceded that in matters of therapeutics it is always difficult to appreciate correctly the relation of cause and effect, and to eliminate the factor of accidents in estimating the efficiency of any plan of treatment. And in order to arrive at a positive and unbiased decision, it is necessary to resort to a series of observations and control experiments of so great an extent that the single observer, even though he have at his disposal a vast amount of material, is only capable of furnishing a small contribution in the discussion of these questions. Furthermore, a certain amount of latitude must always be allowed to individual judgment.

Yet while fully conscious of these limitations I think I am justified in asserting that in my therapeutic trials with Pepto-Mangan I obtained all that can be rationally demanded. And I further consider myself warranted in stating that in view of the unquestionable necessity of ferruginous medication in certain troublesome constitutional affections this preparation acts as a most efficient and useful auxiliary to our therapeutic efforts.—*Therapeutische Monatshefte*, May, 1897.

